# *Pseudocercospora fijiensis* Conidial Germination Is Dominated by Pathogenicity Factors and Effectors

**DOI:** 10.3390/jof9100970

**Published:** 2023-09-27

**Authors:** Karla Gisel Carreón-Anguiano, Rufino Gómez-Tah, Efren Pech-Balan, Gemaly Elisama Ek-Hernández, César De los Santos-Briones, Ignacio Islas-Flores, Blondy Canto-Canché

**Affiliations:** 1 Unidad de Biotecnología, Centro de Investigación Científica de Yucatán, A.C., Calle 43 No. 130 x 32 y 34, Colonia Chuburná de Hidalgo, Mérida C.P. 97205, Yucatán, Mexico; carreon.gisel@gmail.com (K.G.C.-A.); rufino26gt@gmail.com (R.G.-T.); epechb@gmail.com (E.P.-B.); gemalyek@gmail.com (G.E.E.-H.); cdlsantosb@gmail.com (C.D.l.S.-B.); 2 Unidad de Bioquímica y Biología Molecular de Plantas, Centro de Investigación Científica de Yucatán, A.C., Calle 43 No. 130 x 32 y 34, Colonia Chuburná de Hidalgo, Mérida C.P. 97205, Yucatán, Mexico; ignislas@gmail.com

**Keywords:** *Pseudocercospora fijiensis*, black Sigatoka, germinating conidia, effector families, RXLR, LysM, Y/F/WxC

## Abstract

Conidia play a vital role in the survival and rapid spread of fungi. Many biological processes of conidia, such as adhesion, signal transduction, the regulation of oxidative stress, and autophagy, have been well studied. In contrast, the contribution of pathogenicity factors during the development of conidia in fungal phytopathogens has been poorly investigated. To date, few reports have centered on the pathogenicity functions of fungal phytopathogen conidia. *Pseudocercospora fijiensis* is a hemibiotrophic fungus and the causal agent of the black Sigatoka disease in bananas and plantains. Here, a conidial transcriptome of *P. fijiensis* was characterized computationally. Carbohydrates, amino acids, and lipid metabolisms presented the highest number of annotations in Gene Ontology. Common conidial functions were found, but interestingly, pathogenicity factors and effectors were also identified. Upon analysis of the resulting proteins against the Pathogen–Host Interaction (PHI) database, 754 hits were identified. WideEffHunter and EffHunter effector predictors identified 618 effectors, 265 of them were shared with the PHI database. A total of 1107 conidial functions devoted to pathogenesis were found after our analysis. Regarding the conidial effectorome, it was found to comprise 40 canonical and 578 non-canonical effectors. Effectorome characterization revealed that RXLR, LysM, and Y/F/WxC are the largest effector families in the *P. fijiensis* conidial effectorome. Gene Ontology classification suggests that they are involved in many biological processes and metabolisms, expanding our current knowledge of fungal effectors.

## 1. Introduction

Asexual spores or conidia are common structures for the dispersal and perpetuity of most filamentous fungi. Conidiogenesis and conidial germination have been extensively studied and many genes related to these biological processes have been described. For example, in *Neurospora crassa*, the zinc-finger proteins WC-1 and WC-2 regulate conidiogenesis in response to light stimuli [1] and the transcription factors FRQ, FRH, and CK-1 in response to the circadian cycle [2]. Environmental factors such as CO_2_ level, temperature, and desiccation also determine conidial production. The proteins ACON-2, ACON-3, and FLD interrupt filamentous growth and encourage the formation of proconidia [1]. Both conidiogenesis and conidial germination involve autophagy, a catabolic process that recycles cell contents for cell growth and differentiation [3].

Once mature, conidia are dispersed by wind and rain. In phytopathogenic fungi, after contact with the host hydrophobic surface and hydration, conidia rapidly germinate forming a polarized germ tube that secretes mucilage to tightly stick to the surface. Conidial surface proteins, such as hydrophobins and lectins, also play key roles in adhesion, both in mammal and in plant pathogens [4,5,6]. Conidial surface proteins also play additional roles in pathogenesis. In *Metarhizium anisopliae*, an entomopathogenic fungus, conidial surface hydrolases such as protease, trehalase, chitinase, lipase, peroxidase, superoxide dismutase, and phospholipase C are key to the colonization of host insects [7].

The biological processes of conidiogenesis, conidial germination, and appressorium development have been extensively studied, but reports on molecular mechanisms explaining the role of fungal conidia in pathogenicity are still scarce, although it is evident that a relationship exists between conidia and pathogen virulence. One of the earliest studies of the high-throughput identification of pathogenicity factors in conidia was conducted on *Aspergillus fumigatus* by Teutschbein et al. (2010) [8]. They identified by proteomics 225 proteins that had not been previously reported in the *A. fumigatus* mycelial proteome. Interestingly, 57 proteins, including the 40 most abundant proteins, were related to defense and virulence, e.g., spore-specific catalase CatA, bifunctional catalase-peroxidase Cat2, conidial hydrophobin Hyp1, peptidyl-prolyl cis-trans isomerase, Asp hemolysin-like protein, zinc-binding oxidoreductase, Cu-Zn superoxide dismutase, transketolase TktA, and ThiJ/PfpI family protein, among others.

In addition to the concepts of “pathogenicity” and “virulence factor”, the term “effector” has been incorporated into the research terminology regarding pathogen–host interactions, and, to date, effectoromics represents an important line of research in plant pathology [9,10,11], as these molecules play roles in fungal cell protection, the prevention of host perception, and the suppression of a host immune response, among others [12,13].

Many reports on fungal effectors stem from interactions between the pathogen and host; these reports have revealed effectors during appresorium formation and development [9,14,15,16], in biotrophy [17,18,19], and in necrotrophy stages [20,21,22,23,24]. It is known that many effectors are specific to a particular stage during the pathogen–host interaction. However, few reports have focused on the effectors of fungal conidia. In the fungal phytopathogen *Verticillium dahliae*, the effector *Vdpf*, a Zn(II)2Cys6 fungal-specific transcription factor, was found with the highest expression during conidia formation. The deletion of this gene resulted in a reduction of conidial production and the reduced virulence of the fungus in cotton [25].

In *Fusarium oxysporum* f. sp. *cubense* race 4 (Foc TR4), a pathogen that threatens banana production, Hou et al. (2018) [26] reported that the effector SGE1 (Six Gene Expression 1) regulates conidiation and pathogenicity. This gene was also found to be key in *F. oxysporum* race 1 and different *forma specialis* of *F. oxysporum* during infection of their respective host plants [27]. In *F. oxysporum* f. sp. *conglutinans*, multiple effectors were identified in one dispensable chromosome; effectors were named HSs because their mutants are hygromycin B-sensitive. While H2, H3, and H6 effectors regulate conidia production and virulence in Arabidopsis and cabbage hosts, the H5 mutant is virulent on cabbage but not on Arabidopsis [28], evidencing that the roles of conidial effectors are complex, and our current knowledge is still limited. As mentioned before, there are many reports regarding fungal effectors, and most of those reports studied middle- or late-stage host–pathogen interaction. If only a few reports have investigated the molecular basis of pathogenicity in fungal conidia, far fewer have paid attention to their effectors.

The first high throughput analysis identifying effectors in fungal conidia was from Mehta et al. in *Colletotrichum gloeosporioides* [29]. They analyzed the transcriptome during the development of the conidial germ tube and the anastomosis tube, and identified 776 effector candidates in *C. gloeosporioides* conidia, 102 of them being specifically expressed in conidia when compared with mycelia. Pectin lyase, glycosyl hydrolases, mandelate racemase/muconate lactonizing enzyme, peptidase S41, glucanase, among others, were identified among the effector candidates. In the anastomosis tubes, they observed an upregulation in genes involved in oxidative stress responses.

*Pseudocercospora fijiensis* is the causal agent of black Sigatoka disease, one of the most devastating diseases in banana and plantain plantations. Here, we describe a data mining analysis for pathogenicity factors and effectors in transcripts expressed in *P. fijiensis* conidia during germination. Effectors were identified with the WideEffHunter algorithm, resulting in the prediction of 618 effectors. Canonical ones were extracted with EffHunter, resulting in 40 of them in total. While mining the transcriptome for PHI-homologs, 754 hits were retrieved; 225 hits were shared among it, evidencing that more than a thousand conidial functions are dedicated to pathogenicity activities. The most prominent set of effectors is the RXLR family, which was thought to be exclusive to oomycetes until it was recently discovered in fungi as well [30], although their roles in fungi remain to be discovered. The second largest group is the LysM domain family. This domain is responsible for carbohydrate binding and is involved in cell wall protection and remodeling, glycan degradation, and the prevention of host perception of oligomers released by pathogen cell wall damage [31,32]. The third largest family is Y/F/WxC, belonging to a large family of effectors described in the obligate biotrophic fungus *Blumeria graminis* f. sp. *hordei*. This suggests that the conidial effectorome in the hemibiotroph *P. fijiensis* is dominated by functions related to the prevention of host detection, interference with host cell signal transduction, and the suppression of host defense, all prominent effector functions of biotrophic pathogens [33]. Novel discoveries about effectors continue to shape the effectoromics landscape; our results contribute to widening the knowledge about *P. fijiensis* and they also encourage further research on pathogenicity in the conidia of phytopathogenic fungi.

## 2. Materials and Methods

### 2.1. Biological Material and Preparation

The *P. fijiensis* strain C1233 was used in this study. A sample of mycelium was taken from a culture in PDB liquid medium (Difco, Le Pont de Claix, France) and inoculated in 250 mL flasks with 100 mL of PDB liquid medium and cultivated while shaking at 110 rpm for 15 days, with a 12 h photoperiod, at 24 °C (±2). The biomass was collected with sterile gauze and macerated with a mortar and pestle. A 3 mL quantity of the suspension was subcultured in new PDB medium for an additional 15 days. Mycelium was collected and macerated, as above, with 50 mL of PDB medium until homogeneous fragments were obtained, and 2.5 mL of this suspension was plated on Petri dishes with PDA medium (Difco, Le Pont de Claix, France). Cultures were stored at 24 °C (±2) with constant light for 15 days to induce conidiation according to Leyva-Mora et al. [34]. Conidia were collected in 2.5 mL of PDB medium with a sterile brush and filtered with sterile gauze to remove mycelial fragments. To induce conidial germination, 1 mL of conidial suspension was plated on Petri dishes containing 1 mL of 0.05% TritonX-100. Germinating conidia were collected in triplicate from 0, 8 to 24 h, and at 8 h intervals, and stored at −80 °C for subsequent RNA extraction.

### 2.2. RNA Isolation and Transcriptome Sequencing

Total RNA was extracted from germinating conidia with TRIzol reagent (Invitrogen, Waltham, MA, USA) according to the manufacturer’s instructions. Purity and concentration of total RNA was determined using a Nanodrop spectrophotometer (Thermo scientific, Waltham, MA, USA) at 260/280 and 260/230 nm and gel electrophoresis. RNA was treated with DNAase I (Invitrogen, USA) before it was sent to Research and Testing Laboratories (Lubbock, TX, USA) for sequencing. RNA integrity was checked using a Bioanalyzer chip (Agilent Technologies, Santa Clara, CA, USA). All samples were pooled because the amounts of RNA per sample were insufficient for individual analysis. Single strand sequencing was done on the transcripts using 454 pyrosequencing technology.

### 2.3. Sequence Cleaning and Transcriptome Assembly

The quality of the raw data was assessed using the FastQC v.0.11.5 program (https://www.bioinformatics.babraham.ac.uk/projects/fastqc/) [35]. The FastQ Quality Trimmer v.0.0.14 program was used to trim the ends of low-quality transcripts (Phred ≥ 20). De novo transcriptome assembly was carried out using a short read assembling program Trinity v2.4.0 with default parameters [36]. Trinity processed the RNA-seq reads using three independent software modules, *Inchworm*, *Chrysalis*, and *Butterfly* [36].

The *Pseudocercospora fijiensis* version 2.0 genome was subsequently downloaded from the JGI Genome Portal (https://mycocosm.jgi.doe.gov/Mycfi2/Mycfi2.home.html). Then, the assembled transcripts were aligned to the reference genome using Bowtie2 v.2.3.2 with default parameters [37].

A clustering analysis was implemented with CD-HIT-EST software (https://github.com/weizhongli/cdhit) with a 95% identity threshold applied to define the final sets of unigenes while minimizing redundancy [38]. Then, assembled transcripts were translated to coding protein sequences using TransDecoder version 5.7.0 (http://transdecoder.sourceforge.net/) with proteins of at least 30 amino acids (peptides) selected for further analysis (Haas, BJ. https://github.com/TransDecoder/TransDecoder’).

All peptides were further in silico characterized by amino acid length and number of amino acids (Cys, Ser, Thr, Pro, Lys, Asp, and Try) using a set of Perl scripts [39], while signal peptide prediction was done using SignalP 4.1 software with cutoff >0.5 [40]. Transmembrane helices were analyzed and counted (TMDs) using the TMHMM 2.0 software [41].

### 2.4. Gene Ontology Distribution and Functional Annotation

To infer the biological functions of conidial transcripts, functional annotations were performed using stand-alone BLASTx searches against multiple protein databases such as Uniprot, non-redundant GenBank database, KEGG, and InterProScan [42] using an E-value cutoff of 1 × 10^−5^ and a coverage of 55%.

Gene Ontology (GO) mapping for biological processes (BP), molecular functions (MF), and cellular components (CC) were performed using the translated sequences of assembly transcripts against the GO database and the integrated functional domain database in InterProScan v.86.0 [42,43,44] (https://www.ebi.ac.uk/interpro/).

The following analysis used the WEGO software (https://wego.genomics.cn/, accessed on 3 March 2023) to prepare GO functional classification histograms which displayed the sorted *p*-values for these genes and indicated their significant sample differences [45]. Pathway assignments were carried out using default parameters according to the Kyoto Encyclopedia of Genes and Genomes pathway database (KEGG) (https://www.genome.jp/tools/kofamkoala/, accessed on 1 May 2023) to classify and plot the distribution of KEGG annotations [46].

### 2.5. Identification of Transcripts Involved in Pathogen–Host Interaction

The Pathogen–Host Interaction (PHI) database, which is made up of 8411 genes that are involved in pathogenesis/virulence, was downloaded from http://www.phi-base.org/ [47]. To identify pathogenicity-related expressed genes in *P. fijiensis* conidia, a protein search was conducted against PHI database using a stand-alone BLASTp [48]; cut-off parameters were E-value 1 × 10^6^ and identity ≥50%.

### 2.6. Prediction of Effector Proteins

Effector prediction and filtering were performed using WideEffHunter [49] and EffHunter algorithms [39]. The criteria to identify the canonical effectors were (1) protein length ≤ 400 amino acids, (2) extracellular localization, (3) the presence of a signal peptide, (4) at least 4 cysteine residues, and (5) absence of transmembrane domains.

Putative effectors were classified into families according to the presence of domains and/or motifs, or based on homology with known effectors.

To identify conidial-specific effectors, we compared our sequences of the *P. fijiensis* conidia against the transcriptome of the in vitro-cultured *P. fijiensis* mycelia and the transcriptome of *P. fijiensis* during infection with *Musa acuminata* from Noar and Daub [50]; these public transcriptomes (accession SRP075820) were downloaded from http://www.ncbi.nlm.nih.gov/sra/SRP075820 (accessed on 5 December 2022). Effector sequences were used as queries using the stand-alone BLASTp [48]. Those assembly transcripts with no matches were classified as conidial-specific effectors.

### 2.7. Differential Expression of Genes (DEGs) of Top Conidial Transcripts

The top 10 most expressed genes in the conidial transcriptome, plus the top 10 most expressed genes with identified homologs in the PHI database, plus the top 10 most expressed genes identified as effectors were pooled and redundancy was eliminated. The list of “top conidial genes” consisted of 24 genes. The differential gene expression of the top conidial transcripts was analyzed by comparing their expression in conidia, in mycelia, and during infection with *Musa acuminata* using the transcriptome data described above.

The assembled reads were used to estimate relative gene expression. After the assembly, transcripts were mapped, and the RPKM values (Reads per Kilobase per million mapped reads) were calculated and transformed into Z-score [51]. Row clustering was applied to illustrate the expression patterns observed in the data, using the Average Linkage method and the Pearson correlation coefficient for distance measurement between rows and columns (coefficient between 0 and 1). These values were introduced to facilitate the relative comparison of transcript levels between each gene in the transcriptome. The Heatmapper program (http://www.heatmapper.ca/, accessed on 10 September 2023) was used for plotting the Z-score of the relative expression values [52].

## 3. Results

### 3.1. Conidia Germination

The first attempt at conidial germination was done in Petri dishes with drops of liquid PDB medium. Germination occurred very quickly, and after 120 min, conidia were fully germinated (data not shown).

**Figure 1:**
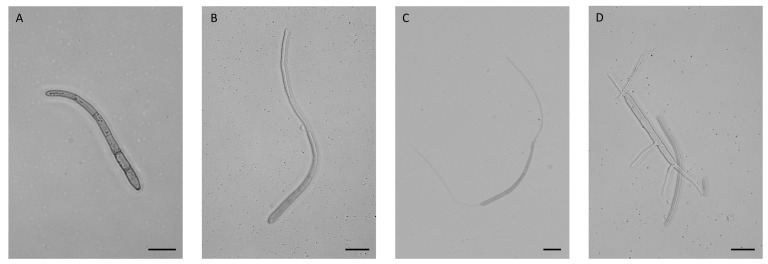
*Pseudocercospora fijiensis* conidia during germination observed at different times. (**A**) 0 h, non-germinated conidia with defined septa and apexes; (**B**) 8 h, Conidia with hyphae developed at the apical apex; (**C**) 16 h, Conidia with hyphae developed in both apexes; (**D**) 24 h, conidia with developed hyphae at the septa and apexes. Conidia were observed under an optical microscope (Nikon Eclipse E200)—Scale bars = 10 µm.

The conidia were then germinated in 0.05% (*v*/*v*) triton in water, pipetting drops of this solution into Petri dishes. The germination of the conidia was much slower here, taking 24 h to occur. Germination was monitored, noting the moments in which most of the conidia looked phenotypically similar. Figure 1 shows *P. fijiensis* conidia during germination. Unfortunately, even with the presence of the detergent, part of the conidia adhered to the Petri dish and the RNA recovered from samples collected at different times was not sufficient to develop independent transcriptomes. RNAs of all three replicates per collection time (0, 8, 12, 16, and 24 h) were pooled into a single sample for sequencing.

### 3.2. Sequence Quality Analysis, Assembly, and Functional Annotation

The transcriptome comprises a total of 23,429 sequences with lengths of 40–72 nucleotides, and a G/C content of 53%. A Phred value of >20 was employed to continue the analyses. According to the FastQC analysis, nucleotides at positions 500–639 had no reliable values. After being trimmed, 489 sequences were eliminated (2%), leaving 22,940 sequences of good quality.

De novo assembly was carried out with the Trinity program (version v2.4.0) using k-mer = 25. As a result, 2501 contigs were generated. These 2501 contigs were aligned against the *P. fijiensis* genome; 96.92% mapped while 3.08% did not align. Table 1 is a summary of these results.

**Figure 2:**
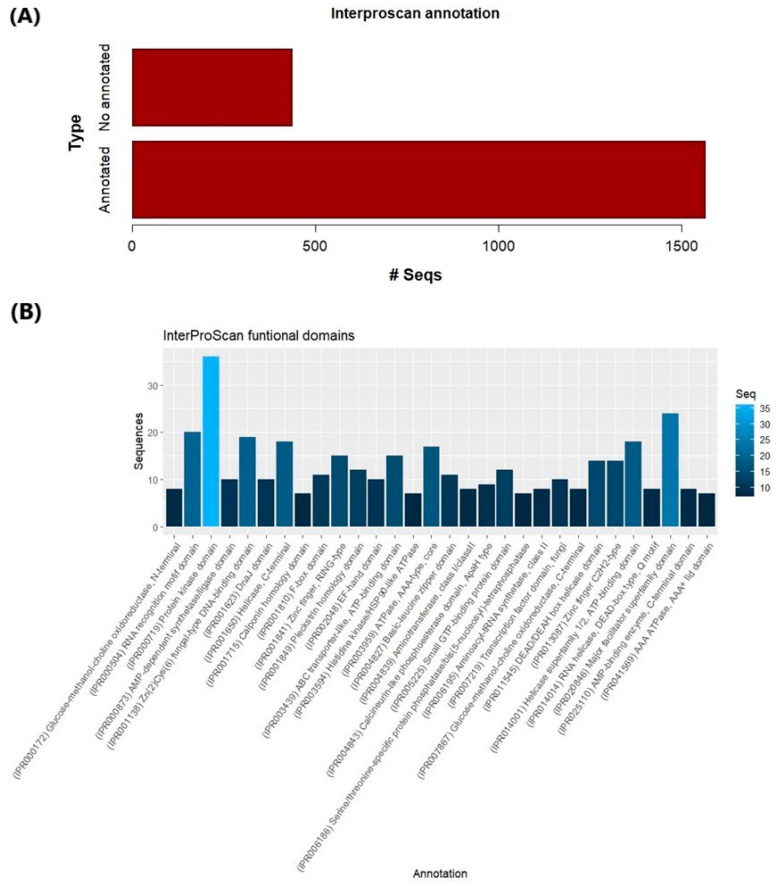
Functional domains in the deduced proteome of *P. fijiensis* conidia during germination. (**A**) Summary of functional domains. (**B**) Histogram of the top 30 InterPro domains of the *P. fijiensis* conidia in germination.

Contigs which mapped with the *P. fijiensis* genome were used as queries to download the complete sequences of the proteins encoded in the transcriptome. Conserved domains were searched for using InterProScan, and a resulting 1564 proteins were found to have domains while 437 lacked domains (Figure 2A). The most frequent domains were protein Kinase IPR000719 (36 genes), major facilitator superfamily IPR020846 (24 genes), RNA recognition motif IPR000504 (20 genes), Zn(2)Cys(6) fungal-type DNA-binding IPR001138 (19 genes), helicase superfamily 1/2, and ATP-binding IPR014001 (18 genes) (Figure 2B).

**Figure 3:**
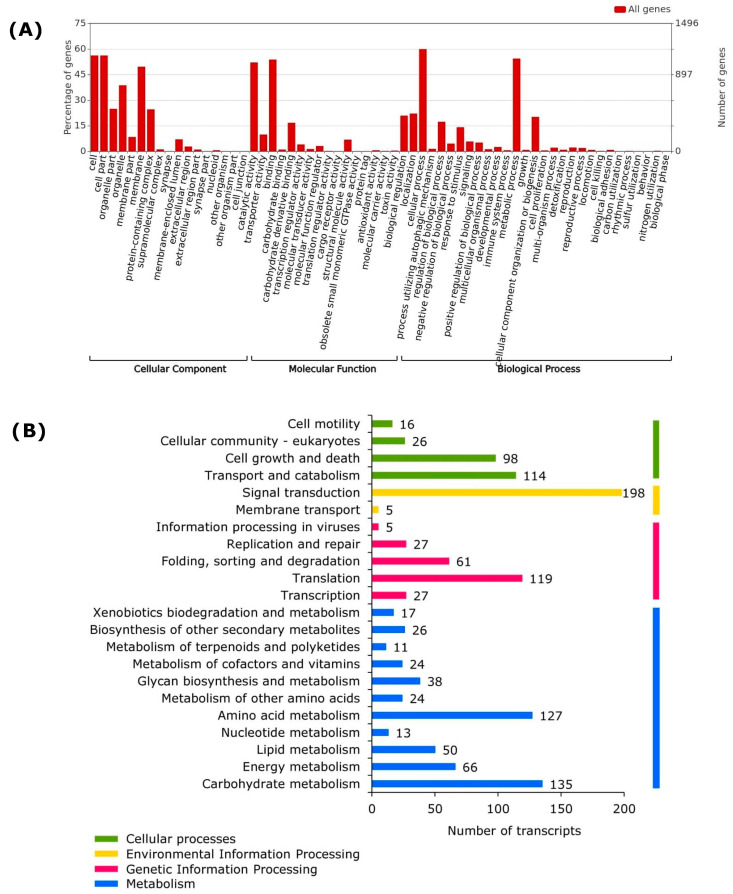
(**A**) Gene Ontology classification of the transcriptome of *P. fijiensis* conidia in germination. The plot corresponds to the most represented functions in the Gene ontology analysis of 2424 contigs that aligned with the *P. fijiensis* genome. (**B**) KEGG pathway enrichment analysis of the transcriptome of *P. fijiensis* conidia in germination.

To calculate the number and percentage of proteins belonging to each GO category, i.e., “molecular function”, “cellular component”, and “biological process”, the gene ontology assignments of the 2424 proteins whose genes were aligned with the *P. fijiensis* genome were submitted to WEGO analysis. A total of 2260 GO annotations were retrieved and distributed as 772 related to a “biological process”, 364 for a “cellular component”, and 1124 for a “molecular function. GO analysis identified 1373 gene products and the most represented GO terms were “cellular process”, “cell part”, “metabolic process”, “binding”, and “catalytic activity” (Figure 3A).

### 3.3. Transcriptional and Translational Activities

The transcriptome shows that metabolism is highly active during conidial germination in *P. fijiensis* (Figure 3A,B). More than 50% of annotated genes are classified as having catalytic activity in the “Molecular Function” (Figure 3A), with hydrolases as the largest group (454), comprising different classes of peptidases, thiol-dependent ubiquitin-specific proteases, lipases, lipid and carbohydrate esterases, different classes of nucleotidases, GTP cyclohydrolase, DNA helicases, enzymes involved in RNA metabolism, and enzymes involved in purine biosynthesis (Table S1).

Three classes of RNA polymerases (I, II, and III) were annotated, as well as the RNA lariat debranching enzyme, key for mRNA maturation. A number of transcription factors were present in *P. fijiensis* conidia, such as the largest transcription factor family Zn2C6, as well as members of the rare family C2H2 [53]; the general transcription factor TFIIB, essential for the formation of the RNA polymerase I initiation complex [54], RNA polymerase II initiation complex [55], and RNA polymerase III initiation complex [56]; AP-1, a bZIP transcription factor that regulates gene expression in response to a variety of stimuli; transcription factor SFL1, involved in cell-surface assembly; transcription factor CRZ1, essential for conidation and virulence; transcription factor GATA, a four-cysteine Zn finger transcription factor that binds to the DNA sequence GATA; and nine pH-response transcription factors (Table S1). These factors regulate nitrogen metabolism and siderophore biosynthesis, among others [57].

### 3.4. Most Active Pathways

Carbohydrate and amino acid metabolisms were the processes found in *P. fijiensis* conidia with the largest number of transcripts (Figure 3B). Transcripts of the pathways Embden–Meyerhof (EM) (glyceraldehyde-3-phosphate dehydrogenase, enolase, fructose-bisphosphate aldolase, glyceraldehyde 3-phosphate dehydrogenase, hexokinases, and pyruvate kinase), pentose phosphate (PP) (6-phosphogluconate dehydrogenase, glucose-6-phosphate dehydrogenase, transaldolase, and transketolase), the TCA cycle (aconitase, citrate synthase, fumarase, isocitrate dehydrogenase, malate dehydrogenase, and pyruvate oxidoreductase), the methylcitrate cycle (isocitrate lyase), mannitol metabolism (mannitol dehydrogenase and mannitol-1-phosphate 5-dehydrogenase), glycogen metabolism (glycogen phosphorylase), and trehalose metabolisms (trehalase) were expressed in *P. fijiensis* spores (Table S1).

Regarding amino acid metabolisms (Figure 3B, Table S1), the biosynthesis pathways for α-ketoglutarates: glutamate (glutamate synthase), glutamine (glutamine synthetase), proline (L-ornithine:2-oxoglutarate 5-aminotransferase, N-acetylglutamate synthase, and N-acetyltransferase), and arginine (argininosuccinate synthase); aromatic amino acids: phenylalanine and tyrosine (chorismate synthase and 4-amino-4-deoxychorismate synthase), and tryptophan (anthranilate synthase); the oxaloacetate/aspartate family: lysine (aspartokinase/homoserine dehydrogenase and aspartate-semialdehyde dehydrogenase), threonine (homoserine dehydrogenase and threonine synthase), methionine (cystathionine beta-synthase and cystathionine-β-lyase), asparagine (aspartate transaminase and asparagine synthase), and isoleucine (acetolactate synthase and valine transaminase); derived from 3-phosphoglycerates: serine (phosphoglycerate dehydrogenase) and cysteine (cystathionine beta-synthase, cystathionine beta-lyase, and cystathionine gamma-lyase); and from ribose 5-phosphates: histidine (ATP phosphoribosyltransferase, phosphoribosylaminoimidazolecarboxamide formyltransferase, and imidazoleglycerol-phosphate synthase), were identified in the annotations of the transcriptome.

Lipid metabolism was the third among the main metabolisms found in *P. fijiensis* conidia (Figure 3B, Table S1). Although few fungal-type fatty acid synthases were expressed, more transcripts encoding enzymes related to lipid catabolism were identified, such as lipid oxidation and fatty acid beta-oxidation (fatty acid amide hydrolase, lysosomal acid lipase/cholesteryl ester hydrolase, carnitine acyltransferases, phospholipase/carboxylesterase, lysophospholipase, triacylglycerol lipase/lysophosphatidylethanolamine acyltransferase, and cardiolipin-specific phospholipase) which catalyze the degradation of cardiolipin, a specific mitochondrial phospholipid [58]. Other lipid catabolic enzymes are involved in signal transduction, as described below.

Protein metabolism is highly active in these spores. The number of GO terms related to the translation was high (119 annotations). A number of small (29) and large (43) subunits of ribosomal proteins were annotated (Table S1), as well as t-RNA ligases (tryptophan-tRNA ligase, glycine-tRNA ligase, tyrosine-tRNA ligase, leucine-tRNA ligase, isoleucine-tRNA ligase, arginine-tRNA ligase, methionine-tRNA ligase, and alanine-tRNA ligase, etc.), components of protein folding (endoplasmic reticulum chaperone BiP, molecular chaperones GrpE, and HtpG), N-glycosylation (UDP-N-acetylglucosamine-dolichyl-phosphate N-acetylglucosaminephosphotransferase), protein degradation (E3 ubiquitin-protein ligase, ubiquitin-conjugating enzyme E2, ubiquitin carboxyl-terminal hydrolase MINDY, and ubiquitination network signaling protein AcrB), and amino acid degradation (indoleamine 2 3-dioxygenase, aminoadipate transaminase, serine/threonine/tyrosine kinase, serine/threonine kinase, dihydrolipoyllysine-residue succinyltransferase, phosphoglycerate kinase, and phosphoglycerate mutase).

Table 2 shows the top 10 transcripts expressed in *P. fijiensis* conidia during germination.

### 3.5. Secretome

A total of 194 extracellular proteins were predicted in *P. fijiensis* conidia during germination (Table S1), which is ~10% of the total number of predicted proteins. Eighty-five of the secreted proteins have enzymatic activities, the largest group corresponding to glycosyl hydrolases (GH family). GHs are a widespread group of enzymes, essential to carbohydrate metabolism, either in the processing of N-linked glycoproteins or the degradation of carbohydrate structures [59]. Glycoside hydrolases from families 8, 16, 17, 31, 47, 79, 89, 90, 92, 114, 125, and 128 were identified in the secretome. Proteases were also present in the conidial secretome, such as three different aspartyl proteases and two serine carboxypeptidases. Extracellular lipases included one member of the class 3 lipase and one lysophospholipase. Five multicopper oxidases were found, two of them tyrosinases, associated with the synthesis of melanin [60] which plays roles in pigmentation, redox balance, and UV protection [61]. Other functions in the secretome are related with cell wall remodeling (one alpha-N-acetylglucosaminidase, three glucanosyltransferases, and fourteen chitinases), which is very active when the fungal biomass is increasing [62]. Cell wall degrading enzymes such as a cellulase, one pectinesterase, one β-glucosidase, and two glucose-methanol–choline (GMC) oxidoreductases, which are relevant for lignocellulose degradation, were also found in the transcriptome [63].

### 3.6. Other Important Processes Usually Found in Conidia

Genes related to common processes in germinating conidia were found, including genes associated with adhesion, perception, signal transduction, conidial germination, ergosterol biosynthesis, autophagy, and gene silencing (Table 3 and Table S2).

### 3.7. Pathogenicity-Associated Functions

#### 3.7.1. PHI Homologs

The conidia play multiple roles in the fungal dispersal, survival, and conquest of new habitats. Conidia are also involved in pathogenesis and, as such, are key to infection. The PHI database search retrieved 754 hits in the transcriptome, 84 of them with predicted extracellular functions (Table S1) such as 1,3-beta-glucanosyltransferases, acid and alkaline proteases, carboxypeptidase Y, DnaJ-domain-containing protein, glycoside hydrolases and glycosyltransferases, hydrophobic surface binding protein A, oxalate decarboxylases, peptidyl-prolyl cis-trans isomerase, PR-1-like protein, U4/U6 small nuclear ribonucleoprotein Prp4, and vacuole protein 4. Functional orthologs of Avr4, Ecp2, and Ecp6 were identified among the PHI homologs in the conidial transcriptome. Homologs of cytosolic proteins found in the PHI database include 14-3-3 proteins, 1,3-beta-glucan synthase, oxoglutarate dehydrogenase, 3′,5′-cyclic-nucleotide phosphodiesterase 1, 3-isopropylmalate dehydrogenase, acetyl-CoA C-acyltransferase, 40S and 60S ribosomal proteins, 4-aminobutyrate aminotransferase, 6-phosphogluconate dehydrogenase, acetyl-CoA acetyltransferase, acetyl-CoA synthetases, adenosylhomocysteinase, adenylosuccinate lyase, ADP-ribosylation factors, amino acid permeases, argininosuccinate synthase, ATP-dependent RNA helicases, autophagy-related proteins, C_2_H_2_ finger domain transcription factors, cAMP-dependent protein kinase, cdc proteins, chaperone protein dnaJ, different classes of chitin synthases, chorismate synthase, cutinase transcription factor 1 alpha, dicer-like protein 1, DnaJ 1, mitochondrial, many enzymes involved in dolichol metabolism, FAD oxidoreductases, Ferric/cupric reductases, FKBP peptidyl-prolyl cis-trans isomerase, fungal-specific transcription factor domain-containing proteins, members of different families of glycoside hydrolases, different classes of kinases (e.g., histidine kinase and serine/threonine protein kinase), metallo-dependent phosphatases, a number of MFS multidrug transporters, and a number of hits related with mitochondrial functions, a number of NAD(P)-binding proteins, such as endo-1,4-beta-xylanase and oxidoreductases. In addition, a number of NAD-dependent proteins were identified, such as NADH: ubiquinone reductase and histone deacetylase; non-ribosomal peptide synthetases, polyketide synthase, peptidyl-prolyl cis-trans isomerases, permeases for cytosine/purines, uracil, thiamine; pH-response transcription factors, PLC-like phosphodiesterase, PLP-dependent transferases, a few Pre-mRNA-splicing factors, a number of proteins involved in RNA metabolism such as RNA exonuclease 4 and RNA-binding domain-containing proteins, septins, shikimate kinase, a number of transcription factors, and functions involved in protein degradation, among others.

The 10 functions with the highest expression levels among PHI homologs identified were glucose transporter rco-3 (MYCFIDRAFT 52736), GMC oxidoreductase (MYCFIDRAFT 77759), serine/threonine-protein kinase (MYCFIDRAFT 156715), pyruvate kinase (MYCFIDRAFT 209751), MFS general substrate transporter (MYCFIDRAFT 129014), mitochondrial-processing peptidase (MYCFIDRAFT 87169), pleiotropic drug resistance (ABC) protein (MYCFIDRAFT 57565), the effector extracellular protein 6 (Ecp6) (MYCFIDRAFT 212004), and two beta-lactamases (MYCFIDRAFT 79446 and 135140) (Table 4). The analysis of the full set of PHI-hits (754 proteins) with the EffHunter and WideEffHunter effector predictors identified 265 effectors, (13 canonical and 252 non-canonical), while the remaining 489 proteins were not predicted to be effectors by either effector predictor (Table S3).

#### 3.7.2. Effectorome Prediction

**Figure 4:**
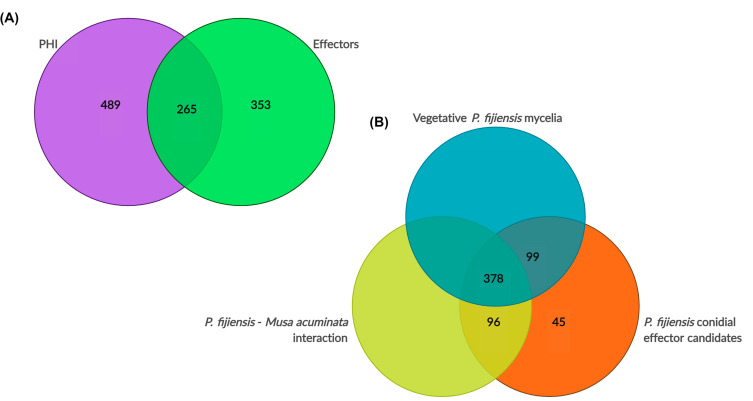
(**A**) Venn diagram depicting functions related to pathogenicity in germinating *P. fijiensis* conidia. Total hits were 1107 (~50% of the transcriptome). Blastp identified 754 pathogenicity/virulence factors with the PHI-database, and WideEffHunter and EffHunter predicted 618 effectors. (**B**) Venn diagram depicting conidial effector candidates that coincide with the transcriptomes of *P. fijiensis* vegetative mycelium and the *P. fijiensis*–banana interaction; both transcriptomes were taken from Noar and Daub (2016) [50]. The number in the orange circle shows the effector candidates which are not supported by the other *P. fijiensis* transcriptomes and are conidially specific.

The effector search retrieved 618 transcriptome hits, with 40 canonical and 578 non-canonical candidates (Table S2). The PHI database shared 265 hits with this effectorome, while 353 had no hits in the PHI database (Figure 4A). Candidate effectors with no hits in the PHI database include, among others, the abscission/NoCut checkpoint regulator, alkaline phosphatase, two cell wall proteins phiA, chaps-domain-containing proteins, proteins involved in vesicle transport such as coatomer, 10 proteins with different DUF domains, extracellular proteins # 7, 21, 52, and 58, histone-lysine N-methyltransferase 2B, monothiol glutaredoxin-7, nascent polypeptide-associated complex subunit beta, phospholipase A2, two RabGAP/TBC, and 48 uncharacterized proteins without any annotation (Table S4).

The ten effector candidates with the highest expression (Table 5) were a mono-oxygenase, two uncharacterized proteins MYCFIDRAFT 212439 and 181747, a putative mitochondrial recombination protein, an oligopeptide transporter, Ecp6, a protein kinase, and ECP2.

Since the conidia were germinated in the presence of triton-X 100, the transcriptome may have reflected the response of the conidia to detergent-imposed stress. To support the contribution of conidial effector candidates to *P. fijiensis* development and pathogenicity, we confirmed the presence of these genes in two previously published transcriptomes: a *P. fijiensis* vegetative mycelium-derived transcriptome and a *P. fijiensis*–banana interaction transcriptome, both publicly available by the authors Noar and Daub [50].

Three hundred and seventy-eight or 61% of the conidial effector candidates were found to be expressed in both transcriptomes [50]. In addition, 99 conidial effector candidates or 16% were shared with the mycelial transcriptome and 96 or 15.5% of the conidial effector candidates were shared with the transcriptome of the *P. fijiensis*–banana interaction (Table S4). Forty-five (45) effector candidates were not supported by the other *P. fijiensis* transcriptomes. However, 18 of these 45 effector candidates have homologs in the PHI database. In summary, 27 effector candidates (4.4%) were not supported by other investigations, but 611 effector candidates (95.6%) were (Figure 4B). In *P. fijiensis*, both structures, conidia and mycelia, are infective structures, and have been used for banana inoculation [64], supporting a probable role for these genes in *P. fijiensis* pathogenesis.

**Figure 5:**
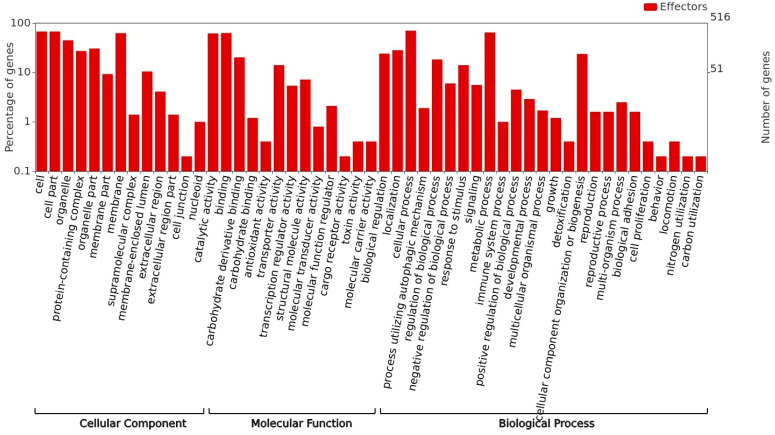
Gene Ontology classification of *P. fijiensis* conidial effectorome. The plot corresponds to 618 effector candidates found in the transcriptome.

Figure 5 shows the Gene Ontology classification of the *P. fijiensis* conidial effectorome. Surprisingly, their functions are not limited to protection and defense, but are involved in all of the processes and metabolisms found in *P. fijiensis* conidia.

**Figure 6:**
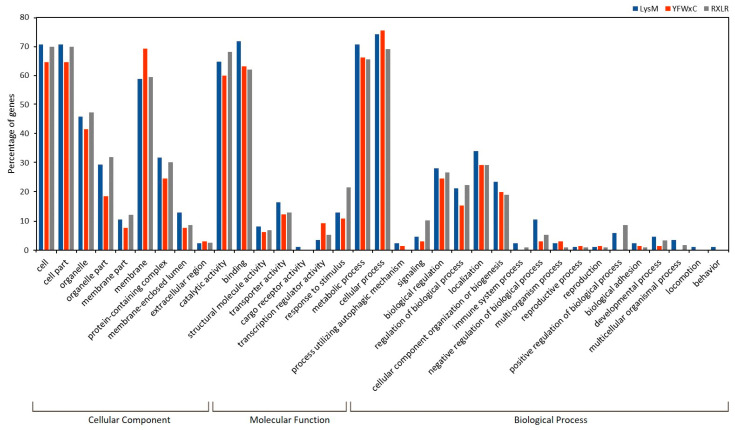
Gene Ontology classification of RXLR, LysM, and Y/F/WxC effector families in *P. fijiensis* conidia.

Conserved effector motifs were identified in the majority of effector candidates (Table S4). A total of 558 contained known motifs, while only 60 (9.7%) had no known motifs. The most frequently identified motifs were: RXLR (161 effector candidates), LysM (100 effector candidates), Y/F/WxC (90 effector candidates), EAR 1 (61 effector candidates), [LI]xAR (60 effector candidates), PDI (25 effector candidates), ToxA (19 effector candidates), crinkler (16 effector candidates), cerato-platanin (three effector candidates), and CFEM (three effector candidates). Figure 6 shows the Gene Ontology classification of the three most frequently identified motifs found in the effectorome, RXLR, LysM, and Y/F/WxC. No specific GO classification was observed among these three families of effectors; rather, they appear to be involved in similar functions, highlighting their role in the metabolic process and regulation within the “Biological Process”, binding and catalytic activity, transport, the regulation of the transcription, and the response to stimuli in the “Molecular function” (Figure 6).

A comparison of the conidial effectorome, with the transcriptome of *P. fijiensis* mycelia grown in culture medium as well as in interactions with the host, as reported by Noar and Daub [50], enabled us to identify conidial specific effectors. In total, 141 conidial effectors were not present in mycelia in vitro and 144 were not shared with the transcriptome of infected banana leafs (Table S4). A comparison of the three transcriptomes identified 46 hits with expression only in the conidia, including one aromatic peroxygenase, one ABC multidrug transporter, one WD40/YVTN repeat-like-containing domain protein, an alkaline phosphatase, an alkaline protease, a homoaconitase, an oligopeptide transporter, a carbamoyl-phosphate, a pyruvate carboxylase, a multidrug efflux transporter, an aldehyde dehydrogenase, one RlpA-like domain protein, and one lysophospholipase, among others.

Two effectors were specific to *P. fijiensis* (Table 6). Table 6 shows the top-10 most expressed conidial transcripts, specific to *P. fijiensis*.

**Figure 7:**
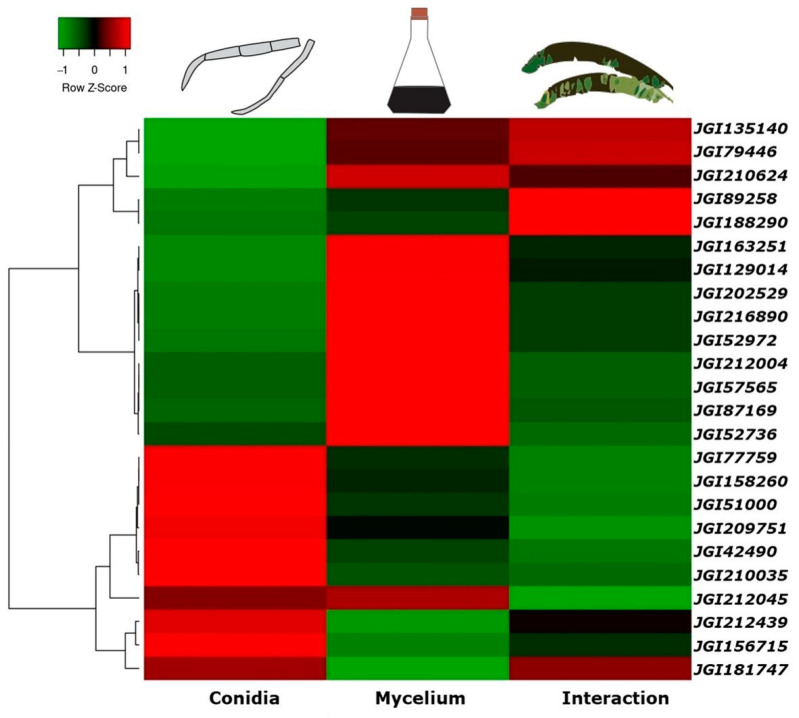
Heat map of top 24 genes in *P. fijiensis* conidia showing differential expression when compared with in vitro-cultured *P. fijiensis* mycelium and *P. fijiensis*–banana interaction. For these two comparisons (mycelium expression and interaction), data were taken from transcriptomes published by Noar and Daub, (2016) [50]. The values represent mean RPKM values transformed into Z-scores. The red color represents the most highly expressed genes, while the green color represents the genes with lowest expression when compared in the different conditions (conidia, mycelium, and interaction). Row clustering was applied to illustrate the expression patters observed in the data. Distinct gene clusters were identified using the Average Linkage method and the Pearson correlation coefficient for distance measurement between rows and columns. The heat map was constructed by plotting the z-score of the relative gene expression values and was generated by analyzing data using the Heatmapper program [52].

To explore how the genes displaying higher expression in conidia were expressed in other conditions, a differential analysis of the genes was performed, comparing their expression in the conidial transcriptome to their expression in the *P. fijiensis* mycelium and *P. fijiensis*–banana interaction, the last two transcriptomes published by Noar and Daub [50]. We combined the top 10 most expressed genes in conidia during germination, the top 10 most expressed genes with homologs in the PHI database, and the top 10 most expressed genes identified as conidial effectors. After elimination of redundancy, the total list was comprised of 24 genes. It is worth noting that all of these genes were expressed in all conditions (conidia, mycelium, and interaction with host). The expression of eight genes (MYCFIDRAFT 77759, 158260, 51000, 209751, 42490, 210035, 212439, and 156715) was highest in conidia; seven of these eight genes are among the list of the top 10 most expressed genes in germinating conidia: GMC oxidation, protein Vip1, thioredoxin-like protein, monooxygenase, K(+)/H(+) antiporter 1, serine/threonine-protein kinase, and one uncharacterized protein (Table 2). Twelve of the most highly expressed conidial genes showed higher expression in the mycelium when this comparison was made, while four of the most expressed genes in the conidia showed higher expression during the interaction with the host (Figure 7).

## 4. Discussion

### 4.1. High Transcriptional Expression Levels

A transcriptomic analysis of germinating *P. fijiensis* conidia showed that *P. fijiensis* is highly active at this stage. Sixteen amino acid biosynthetic pathways were present, as well as the components of protein biosynthetic machinery and t-RNA ligases, revealing that amino acid and protein synthesis are very important processes occurring in *P. fijiensis* conidia during germination.

The pathways for the branched-chain amino acids alanine, valine, and leucine were absent. It is likely that the needs for these amino acids in *P. fijiensis* conidia are met through the process of autophagy. ATG7, ATG8, ATG15, and ATG22 were present in the transcriptome of *P. fijiensis* conidia, and ATG22 is a vacuolar effluxer of leucine and other amino acids resulting from autophagic degradation [65]. Furthermore, ATG8 and ATG15 link autophagy with lipid metabolism [66,67], and ATG15 regulates lipolysis by activating triacylglyceride lipases, which promotes lipid breakdown [67,68]. In *P. fijiensis* conidia, lipid metabolism was the third most annotated in the transcriptome according to the Gene Ontology analysis, but the higher number of transcripts was related to lipid degradation rather than lipid biosynthesis, which shows that the catabolism of lipids is more active than that of lipid biosynthesis. Lipids play an energy storage role in fungal spores and are mobilized for conidial germination and hyphal growth [69,70]. Protein transporters and lipid transporters were identified, consistent with the protein and lipid degradation in *P. fijiensis* conidia.

Consistent with the importance of protein synthesis in *P. fijiensis* conidia, transcriptional activity was also active. Three classes of RNA polymerases (I, II, and III) and the RNA lariat debranching enzyme, key for mRNA maturation, were identified. Several transcription factors were present in *P. fijiensis* conidia such as the commonly described Zn2C6 family, as well as members of the rare family, C2H2 [53], the general transcription factor TFIIB, essential for the formation of the initiation complex for RNA polymerase I [54], RNA polymerase II [55], and RNA polymerase III [56], AP-1, a bZIP transcription factor that regulates gene expression in response to a variety of stimuli, the SFL1 transcription factor, involved in cell-surface assembly, the CRZ1 transcription factor, essential for conidiation and virulence, and the transcription factor GATA, a four-cysteine Zn finger transcription factors that binds to the GATA DNA sequence. These factors regulate the nitrogen metabolism and siderophore biosynthesis, among other transcription factors [57]. Interestingly, pH-response transcription factors were identified in *P. fijiensis* conidia. These factors mediate gene regulation in response to the environmental pH. Burgos-Canul et al. [71] compared the proteomes of two strains of *P. fijiensis* and found, in one of them, the Oz2b strain, the enzymes glutamate decarboxylase, peptidyl-prolyl cis–trans isomerase, adenylosuccinate synthetase, and glutathione synthetase, which are acidic pH-responsive virulence factors. Conversely, in the C1233 strain, urease and an ammonium transporter were found, which are involved in the alkalization of the environment through the excretion of ammonia. Since Oz2b is more virulent than C1233, it was not clear if C1233 and Oz2b follow different pathways of pH regulation to develop infection, or if their proteomes reflect different stages of the infection process. Here, C1233 was used to produce the conidia studied, and the transcriptome was prepared from pooled samples collected at different times of germination. Interestingly, enzymes involved in the regulation of alkalinization and acidification were present, supporting the possibility of a sequential regulation of gene expression under different pHs. Some fungi, such *Colletotrichum gloeosporioides* and *Magnaporthe oryzae*, combine both mechanisms at different stages of infection of their hosts [72].

### 4.2. Storage and Energy Metabolisms

The mycelium of *P. fijiensis* grows very poorly in vitro, as well as in natural infections of the host [73]. The incubation time between infection and the development of symptoms is long (between 1 and 2 months) regardless of the host susceptibility, strain virulence, and environmental conditions [74,75,76]. However, the time between the attachment of the conidia to the leaf surface and germination and entry through the stomata takes 48 to 72 h [77]. According to the rapid germination process of conidia, transcriptomics evidence shows that carbohydrate and protein metabolisms are highly active, as well as the autophagy process of lipids and the uptake of purines and pyrimidines.

The greatest number of transcripts was classified by Gene ontology corresponded to carbohydrate and energy metabolism, including enzymatic activities related to carbohydrate storage compounds (trehalose, mannitol, and glycogen). The carbohydrate metabolism was also the most active pathway in *Aspergillus flavus* and *Aspergillus nidulans* conidial germination [78,79]. Similar results were found by Burgos-Canul et al. [71] in the mycelia of *P. fijiensis*.

More than 50% of the molecular function in Gene Ontology was classified as catalytic activity with hydrolases as the largest group (454), comprising different classes of peptidases, lipases, and different classes of nucleotidases. The largest group of hydrolases is made up of the family of glycosyl hydrolases (GH); GH members found in *P. fijiensis* conidia are involved in various functions including carbohydrate biosynthesis and degradation (e.g., GH47, GH51, GH79, and GH92), cell wall construction or remodeling (e.g., GH16 and GH31), and defense (e.g., GH3 and GH114) [80].

### 4.3. Defense and Oxidative Stress

Defense-related functions were highly active (e.g., the ABC multidrug transporters and MFS drug transporter), as were redox activities (the siderophore biosynthetic process, ferric-chelate reductase, protein disulfide oxidoreductase, catalases, thioredoxin peroxidases, ferredoxin-NADP+ reductase, NADH dehydrogenase, NADPH dehydrogenase, and superoxide dismutases). The presence of these activities is not surprising since *P. fijiensis* is highly tolerant to oxidative stress [81].

Conidia are adapted to adverse conditions such as oxidative stress and UV radiation. *P. fijiensis* conidia are dark because of melanin, which is consistent with the presence of non-reducing polyketide synthase PKS1 [82], as well as highly reducing polyketide synthase alnA, an integral PKS involved in fungal conidiation, antioxidant activity, conidial cell wall integrity for the conidial cell, and UV tolerance [83]. Previously, Noar and Daub [50] reported that polyketide synthases of *P. fjiensis* are more expressed in infected banana leaves compared to mycelia in culture medium, demonstrating that PKSs are also involved in pathogenesis.

### 4.4. Pathogenicity

Although much has been studied about the fundamental biological processes in conidia (biological processes related to production and germination), less attention has been paid to pathogenesis. Considering the PHI homologs and effectors, 1107 functions in the transcriptome are related to pathogenesis and/or virulence, i.e., about 50% of the conidial transcriptome. This shows that pathogenesis dominates the transcriptome of *P. fijiensis* conidia.

The search results from the PHI database and the identification of effectors using WideEffHunter revealed 265 shared hits. However, each search had specific hits: 489 homologs corresponding to PHI and 353 to the effector prediction with WideEffHunter. Therefore, it is recommended that pathogenicity-related functions are searched for using a combination of different tools [84,85].

Usually, the identification of effectors focuses on small, secreted, cysteine-rich proteins. The effectors that meet these structural characteristics are termed “canonical”. Recently, the EffHunter algorithm was created and was shown to have the highest F1 score among fungal effector predictors when identifying these canonical or classical effectors [39]. A novel algorithm with the ability to identify non-canonical fungal effectors was published, WideEffHunter, which simultaneously possesses the ability of EffectorP 1.0 to predict effectors in non-pathogenic organisms, and the added ability of Effector P 2.0 to predict effectors in pathogenic fungi [49]. Here, 40 effector candidates (less than 10% of the effectorome) meet the classical characteristics, and this proportion coincides with WideEffHunter findings when fungal effectoromes were mined [49]. Thirty-five of these canonical effectors were previously predicted in the *P. fijiensis* genome by Arango-Izaza et al. [75]. In this report the criteria used to identify protein effectors were: ≤300 amino acids, ≥4 cysteine residues, and no TMDs. The other five canonical effector candidates in *P. fijiensis* conidia are larger than 300 amino acids, but these five candidates are expressed in the *P. fijiensis*–banana interaction (Table S4). Likewise, Chang et al. [86] identified canonical effectors in *P. fijiensis*, but used ≤250 amino acids as the cut off; 29 of the conidial effector candidates identified here meet this criterion. Interestingly, it was found that non-canonical effectors represent 90% of effectoromes, suggesting that the implementation of non-classical features may be mandatory in fungal effectoromics.

Noar and Daub [50] identified, for first time, candidate *P. fijiensis* pathogenicity genes based on transcriptomic data. Here, we compared the list of *P. fijiensis* conidial effectors with transcriptomes (vegetative mycelia and the *P. fijiensis*–banana interaction) published by these authors and found that 573 were supported by expression in either of these transcriptomes. Forty-five effectors had no match with any of these transcriptomes, but 18 of these 45 putative conidial-specific effectors have homologs in the PHI database. Therefore, only 27 candidates (4.4%) of the whole predicted *P. fijiensis* conidial effectorome were not supported by any other report, while 591 (95.6%) were supported by previously reported transcriptomes. These findings validate our approach for the identification of non-canonical effectors in pathogenicity studies. The other 27 genes may be conidial-specific or stress-responsive genes induced by the triton-X 100.

In *Colletotrichum gloeosporioides* conidia, 776 effector candidates were identified [29], similar to the number of effectors found here in germinating *P. fijiensis* conidia. Similar functions were found between both fungal pathogens: proteins involved in adhesion, enzymes involved in lignin breakdown and cell wall degradation, as well as functions involved in pathogenesis such as GMC oxidoreductase, S41 peptidase, and non-ribosomal peptide synthetase required for pathogenesis and virulence.

When we compared the expression of the 24 most expressed conidial genes, with their expression in *P. fijiensis* mycelium and with the *P. fijiensis*–banana host interaction, eight genes showed maximum expression in conidia. These genes were GMC oxidoreductase, the protein VIP1, a thioredoxin-like protein, a monooxygenase, K(+)/H(+) antiporter 1, a serine/threonine protein kinase, and an uncharacterized protein.

The most highly expressed pathogenicity factors included glucose transporter rco-3 (MYCFIDRAFT 52736), which regulates the affinity of transporters for glucose in order to cope with environmental glucose fluctuations [87], evidencing the hostile and nutrient-limiting environment that the conidia face when they begin to infect the host.

Glucose–methanol–choline (GMC) oxidoreductase was the second hit (MYCFIDRAFT 77759) with the highest expression. GMC oxidoreductase belongs to a large and diverse superfamily, involved in the biocontrol of several plant pathogenic fungi [88]. In *Aspergillus nidulans*, the GMC oxidoreductase is required for the induction of asexual development [89]. The GMC oxidoreductase detected in *P. fijiensis* conidia is predicted to be extracellular. In the hemibiotrophic basidiomycete fungus *Moniliophthora perniciosa*, a methanol oxidase enables the fungus to hydrolyze the methanol released from esterified pectin [90]. Methylesterases are induced upon fungal infection as part of the plant defense mechanism [91,92]. GMC oxidoreductase in *P. fijiensis* may be involved in conidial development and/or the degradation of toxic residues released from the pectin. GMC oxidoreductases are involved in the biosynthesis of secondary metabolites that may play a role in defense against antagonists. The GMC catalyzed conversion of aromatic alcohols is required for patulin synthase (PatE) to catalyze the final step in the biosynthesis of the mycotoxin patulin in *Penicillium expansum* [93].

Serine/threonine (Ser/Thr) protein kinase (MYCFIDRAFT 156715) was the third most expressed protein. The analysis of fungal phosphoproteomes showed that serine and threonine phosphosites are highly represented (~80%), in comparison with tyrosine phosphosites, and they are involved in the regulation of cell wall synthesis, the maintenance of cellular integrity, the cell cycle, transcription, metabolism, as well as virulence [94]. All these functions are important in conidia and congruent with the high expression of a Ser/Thr kinase in *P. fijiensis* conidia. In *Magnaporthe oryzae*, the deletion of MoSch9, a Ser/Thr kinase, prevented the formation of appressoria and conidiophores, leading to the loss of pathogenesis and cold stress tolerance [95].

Pyruvate kinase is an enzyme that catalyzes the conversion of phosphoenolpyruvate and ADP to pyruvate and ATP. In fungi, the accumulation of pyruvate plays an important role in tolerating heat stress through reducing protein carbonylation and stabilizing mitochondrial membrane potential [96,97]. Furthermore, in *Fusarium graminearum*, a pyruvate dehydrogenase kinase 2 (PDK2) is also associated with conidiation, mycelial growth, and pathogenicity [98]. Bananas are cultivated in tropical and subtropical regions worldwide, where solar irradiation and heat are high; thus, in *P. fijiensis*, pyruvate kinase may play roles in protecting conidia against heat, fungal development, and promoting virulence.

Mitochondrial-processing peptidase (MPP) is classified as a M16B subfamily protein in the MEROPS database and is involved in the processing of about 70% of imported precursor proteins [99,100]. MPP is key to many fungal processes, including pathogenicity, since fungal phytopathogens use MPP to infect plant hosts and induce diseases [101]. MPP activity is possibly coordinated with the pyruvate kinase activity since MPP requires ATP. Congruently, the expression levels of both proteins are similar in *P. fijiensis* conidia (rpkm of 351.6 for pyruvate kinase and 333.7 for MPP).

The VIP1 protein (MYCFIDRAFT 158260) is important in the regulation of gene expression and cell signaling [102]. In fungi, it appears to be involved in plant–pathogen interactions, nuclear transport, transcriptional regulation, responses to environmental stress, and antagonistic interactions [103], but its function in conidiation requires further investigation.

The thioredoxin-like proteins (Trx-like proteins) contain conserved cysteine residues that can switch between reduced and oxidized states, allowing them to act as antioxidants in cellular redox homeostasis [104]. Fungal spores are often exposed to environmental stresses, including oxidative stress. In *P. fijiensis*, the Trx-like protein (MYCFIDRAFT 51000) likely scavenges reactive oxygen species (ROS) and protects the conidia from oxidative damage, which is critical for antioxidant function during spore maturation and dormancy [105]. In *Verticillium dahliae,* an extracellular Trx1 mutant (ΔVdTrx1) is unable to eliminate ROS generated by the host during pathogen invasion. The ΔVdTrx1 mutant showed significantly reduced virulence on *Gossypium hirsutum* and in the model plants *Arabidopsis thaliana* and *Nicotiana benthamiana* [106].

Among the highest expressed transcripts are two encoded beta-lactamases (MYCFIDRAFT 79446 and 55784) [107]. *Cladosporium fulvum*, a close relative of *P. fijiensis*, produces the bianthraquinone cladofulvin, whose biosynthetic cluster is composed of 10 genes, including a non-reducing polyketide synthase and a beta-lactamase [108]. Noar et al. [109] identified in *P. fijiensis* the genes involved in the production of the emodin intermediate in the cladofulvin pathway, but they differ in many genes, suggesting the production in *P. fijiensis* of a different lactam metabolic product. In *Fusarium verticillioides*, beta-lactamase confers resistance to the γ-lactam benzoxazinoid (phytoanticipin) produced by maize, wheat, and rye [110]. While searching for metabolic pathways in *Musa acuminata* (wild Malaysian banana) using KEGG (https://www.genome.jp/pathway/mus01100; accessed on 18 June 2023), two entries for lactam compounds were retrieved, C00395 penicillin and C06564 penicillin N, suggesting the production of lactam metabolites in banana. Whether these beta-lactamases are involved in the production of lactam compounds from *P. fijiensis* or in the detoxification of plant lactam compounds is largely unknown, but in either situation the fungus requires a high expression of transporters. Consistent with this, among the most expressed genes are the MFS transporter and the pleiotropic drug resistance (ABC) protein.

Twelve of the top expressed conidial genes of *P. fijiensis* were even more highly expressed in the mycelium, suggesting that these genes are mainly involved in growth and development; the mycelium is also an infective structure [34,64,111,112], so their role in *P. fijiensis* pathogenesis cannot be ruled out. Likewise, four of the highest expressed conidial genes showed a higher expression in *P. fijiensis* in interaction with the host, supporting the hypothesis of pathogenicity roles of these genes.

He et al. [113] pointed out the relevance of identifying conserved amino acid motifs (such as RXLR) in oomycetes to enable the discovery of hundreds of effectors per genome. This motif was long believed to be unique to oomycetes. In *Blumeria graminis* f.sp. *hordei*, Godfrey et al. [114] identified RxLR-dEER and Y/F/WxC motifs in 142 effector candidates for the first time, and recently, using motif-based mining, Zhang et al. [30] identified 635 effector candidates in the *Puccinia triticina* transcriptome in interactions with wheat. The number found in the conidia of *P. fijiensis* is similar (618 effectors). In total, 90% of these effectors have motifs, which is in agreement with the proportion of motif-containing effectors in fungal effectoromes [49]. Interestingly, the most frequently identified motif was RXLR (161 effector candidates), while cerato-platanin and CFEM (motifs known in fungal effectors) were the minority (three for each of them). Similar results were reported in *Puccinia triticina*, with RXLR and CFEM being the largest and the smallest groups, respectively [115]. LysM, a motif ubiquitous in all kingdoms, and Y/F/WxC, a motif originally associated with powdery mildew effectors, were also large families identified in the *P. fijiensis* conidial effectorome (100 and 90 effector candidates, respectively). Motif extraction in a positive data set with 314 true fungal effectors found Y/F/WxC as the most frequent motif [49]. Although the exact function of Y/F/WxC-effectors is unclear, effectors with this domain are predominantly expressed in haustoria and linked to haustoria formation in the obligate biotrophic pathogen *Blumeria graminis* f.sp. *hordei*, and conidia that fail to generate haustoria die [114]. Y/F/WxC effectors in *P. fijiensis* probably play similar roles during the biotrophic stage. In the case of the LysM motif, most LysM effectors prevent the host recognition of chitin through chitin oligomer sequestration [31,32]. The Ecp6 effector (MYCFIDRAFT 212004), which sequesters chitin oligomers and prevents host perception, is among the 10 most expressed effectors in *P. fijiensis* conidia, while Avr4 (MYCFIDRAFT 87167), which protects the cell wall against host chitinases is the 403rd according to its level of expression among the effectors. This evidence suggests that avoiding perception by the host is a very important strategy for *P. fijiensis* in the early stages of its life cycle and probably during the biotrophic stage.

Ranking 23rd in the order of expression among effectors is a permease presumably with purine/cytosine/uracil/uridine/allantoin permease activity, showing that the uptake of foreign purines and pyrimidines is important. On the contrary, two IMP cyclohydrolases, involved in de novo purine biosynthesis, occupy positions 521 and 522; likewise, dihydroorotate dehydrogenase, crucial for pyrimidine biosynthesis, and uridine phosphorylase and uridine kinase, key enzymes in the pyrimidine salvage pathway, occupy positions 1698, 447, and 901, respectively. This transcriptional profile suggests that in *P. fijiensis* conidia, the acquisition of foreign nucleotides plays a more important role than the biosynthesis and salvage pathways. This is unusual in fungi, in which, in many of them, biosynthesis is crucial during vegetative and infective growth [116,117,118]. In fact, new fungicides targeting purine or pyrimidine biosynthesis are being designed [119,120,121]. However, the relevance of each pathway varies in microorganisms; for example, *Candida albicans* and *Schizosaccharomyces pombe* can use purines as nitrogen sources, while *Saccharomyces cerevisiae* cannot because it lacks enzymes involved in purine oxidation [122]. Like other contrasting examples, in *Verticillium dahliae*, the mutation of the VdTHI20 gene, involved in the biosynthesis of pyrimidines, prevents the germination of conidia [123]. In *Uromyces phaseoli*, the purine, the pyrimidine ratio decreased in 8 h from 1:34 to 1:02 because pyrimidine biosynthesis was inactive or very low while the transcription activity was high during uredospores germination [124].

### 4.5. Conidial Processes Regulation by Gene Silencing

Gene silencing is a conserved eukaryotic homology-dependent post-transcriptional mechanism of gene expression regulation. In fungi, gene silencing controls growth, development, and virulence [125]. Gene ontology found an upregulation of gene silencing by miRNA. In addition to the regulation of endogenous processes, the presence of clathrin transcripts and clathrin adapters opens the possibility of an exogenous dsRNA uptake via endocytosis [126], while the presence of coatomers may suggest the export of dsRNA by exosomes [127]. The silencing machinery is very active during conidial germination, probably for the regulation of conidial processes, but defense and virulence functions cannot be ruled out.

### 4.6. Transcriptome Representation

The conidia of *P. fijiensis* were germinated in drops of water with triton detergent on Petri dishes (hydrophobic surfaces), mimicking the surface of the host, and as expected, the conidia behaved in a similar manner as when the host is detected. The known effectors Ecp6 (MYCFIDRAFT 212004) and Ecp2 (MYCFIDRAFT 52972) were among the 10 most expressed effectors; the former sequesters chitin oligosaccharides are released from the cell walls of invading hyphae to avoid perception by the host. Consistent with a behavior similar to the interaction with its host, the transcriptome revealed proteins involved in hydrophobic adhesion, perception, signal transduction, and the expression of a number of cell-wall-degrading enzymes relevant to lignocellulose degradation, such as GMC oxidoreductase, pectin esterase, cellulase, cutinase, among others.

The transcriptional activities found here (carbohydrate, amino acid metabolisms, containment of oxidative stress, autophagy, the number of PHI-database homologs, etc.) are consistent with the results reported in the conidia of other fungi [3,29,79,128]. Moreover, 23 of 37 proteins identified as conidial-specific in the *Aspergillus fumigatus* proteome [8] were encoded by *P. fijiensis* conidial transcripts, reinforcing that this transcriptome reflects the conidial biology of this pathogen. It is worth mentioning that, although the transcriptome sequenced here was a small one (23,429 reads; 2501 contigs), it enabled us to discover new and interesting conidial functions related to pathogenicity. Furthermore, this is the first of its kind to reveal fungal non-canonical effectors, including RXLR and Y/F/WxC families, which were unexpected in conidia, uncovering novel insights in the conidial biology of fungal pathogens.

## 5. Conclusions

Pathogenicity is not a secondary function in *P. fijiensis* conidia, but plays a primary role in conquering the plant host. Around 50% of the transcriptome is devoted to pathogenicity functions. The fungus appears to spend much of its energy avoiding perception by the host, which explains why the plant launches its defense response very late during the infection [129,130,131]. The *P. fijiensis* conidial effectorome is dominated by the RXLR, LysM, and Y/F/WxC families, which appear to play unexpected and redundant metabolic roles according to the Gene Ontology classification. These results expand our current knowledge of the roles of fungal effectors.

## Figures and Tables

**Table 1:** Summary of the transcriptome of *Pseudocercospora fijiensis* conidia during germination.

Read Statistics	# seqs
Raw reads	23,429
Clean reads	22,940
Total contigs	2501
Total mapped	2424
GC content	53%
Sequence repetition (%)	85.16%

**Table 2:** Top 10 most expressed genes in *P. fijiensis* conidia during germination.

JGI ID	NCBI Description	GO Term	RPKM
210035	K(+)/H(+) antiporter 1	GO:0015297 GO:0016020 GO:1902600	555.70
158260	Protein vip1	GO:0003723 GO:0004408 GO:0004843 GO:0005743 GO:0006511 GO:0016579 GO:0046872	555.70
52736	Putative glucose transporter rco-3	GO:0005829 GO:0008643 GO:0009086 GO:0015144 GO:0015295 GO:0016020 GO:0035999 GO:0055085 GO:0071949 GO:0106312 GO:0106313	431.76
77759	GMC oxidoreductase	GO:0016614 GO:0050660	417.66
42490	Mono-oxygenase	GO:0004499 GO:0050660 GO:0050661	412.41
156715	Serine/threonine-protein kinase	GO:0000781 GO:0000922 GO:0000939 GO:0004674 GO:0005524 GO:0005813 GO:0005814 GO:0005876 GO:0006468 GO:0007052 GO:0010971 GO:0032133 GO:0033316 GO:0034501 GO:0034503 GO:0035175 GO:0090148 GO:0090267 GO:0120110 GO:0140429 GO:0140602 GO:1902412 GO:1903380 GO:1904967 GO:1905824 GO:1990023 GO:1990385	395.61
216890	Fluconazole resistance protein 1	GO:0000981 GO:0006357 GO:0008270	388.58
51000	Thioredoxin-like protein	GO:0003756 GO:0005773 GO:0005783 GO:0005886	386.86
212439	Uncharacterized protein	GO:0016020	364.29
212045	FAD-linked oxidoreductase sorD	GO:0016020 GO:0016491 GO:0071949 GO:1901360	359.30

**Table 3:** Important processes found in the *P. fijiensis* conidial transcriptome.

Processes	Genes Annotated	RPKM
Adhesion, perception, and signal transduction	59	16.35–395.61
Conidial germination	71	20.87–266.56
Ergosterol biosynthesis	10	82.63–224.76
Glycosyl hydrolases (secreted)	16	44.47–158.96
Autophagy	22	42.40–224.18
Gene silencing	9	91.84–166.85

**Table 4:** Top 10 most expressed genes with PHI homologs in *P. fijiensis* conidia during germination.

JGI ID	Homolog in PHI	PHI Description	GO Term	RPKM
52736	*Magnaporthe oryzae* (G4N740)	Unaffected pathogenicity	GO:0005829 GO:0008643 GO:0009086 GO:0015144 GO:0015295 GO:0016020 GO:0035999 GO:0055085 GO:0071949 GO:0106312 GO:0106313	431.76
77759	*Brucella abortus* (Q2YIV2)	Reduced virulence	GO:0016614 GO:0050660	417.66
156715	*Fusarium graminearum* (V6RFV4)	Lethal	GO:0000781 GO:0000922 GO:0000939 GO:0004674 GO:0005524 GO:0005813 GO:0005814 GO:0005876 GO:0006468 GO:0007052 GO:0010971 GO:0032133 GO:0033316 GO:0034501 GO:0034503 GO:0035175 GO:0090148 GO:0090267 GO:0120110 GO:0140429 GO:0140602 GO:1902412 GO:1903380 GO:1904967 GO:1905824 GO:1990023 GO:1990385	395.61
209751	*Magnaporthe oryzae* (G4MXS1)	Unaffected pathogenicity	GO:0000050 GO:0000287 GO:0004053 GO:0004743 GO:0005524 GO:0005737 GO:0006091 GO:0006525 GO:0016301 GO:0016310 GO:0016491 GO:0016836 GO:0016866 GO:0030955 GO:0042866 GO:0046031 GO:0046034 GO:0061621	351.60
129014	*Parastagonospora nodorum* (Q6Y392)	Unaffected pathogenicity	GO:0016020 GO:0035442 GO:0071916	346.95
87169	*Helicobacter pylori* (O25656)	Reduced virulence	GO:0004222 GO:0006627 GO:0016020 GO:0017087 GO:0044237 GO:0046872	333.70
57565	*Botrytis cinerea* (Q9UW03)	Unaffected pathogenicity	GO:0005524 GO:0015288 GO:0016887 GO:0034219 GO:0046930 GO:0140359	296.71
212004	*Fulvia fulva * (B3VBK9)	Effector	-	294.05
79446	*Xanthomonas campestris* (Q4UWM4)	Reduced virulence	GO:0016020 GO:0031411 GO:0043227	282.64
135140	*Cryptococcus neoformans* (Q874K8)	Reduced virulence	GO:0003735 GO:0004585 GO:0005247 GO:0005769 GO:0005773 GO:0005794 GO:0005840 GO:0005886 GO:0006412 GO:0015078 GO:0015297 GO:0016597 GO:0016787 GO:0019240 GO:0022853 GO:0042450 GO:1902476 GO:1990904	269.85

**Table 5:** Top 10 most expressed effector genes in *P. fijiensis* conidia during germination.

JGI ID	NCBI Description	GO Term	RPKM
42490	Mono-oxygenase	GO:0004499 GO:0050660 GO:0050661	412.41
212439	Uncharacterized protein	GO:0016020	364.29
88290	Putative mitochondrial recombination protein	GO:0000002 GO:0000150 GO:0003697 GO:0003735 GO:0004553 GO:0005634 GO:0005762 GO:0005975 GO:0006261 GO:0006310 GO:0010557 GO:0016020 GO:0031328 GO:0045740 GO:0071897	349.72
202529	Oligopeptide transporter	GO:0000329 GO:0035672 GO:0035673	313.75
181747	Uncharacterized protein	-	303.58
212004	Extracellular protein 6	-	294.05
210624	Kinase-like protein	GO:0004672 GO:0005524 GO:0006468	266.56
163251	Ferric/cupric reductase transmembrane component 7	GO:0000293 GO:0005886 GO:0006826 GO:0006879 GO:0009987 GO:0065008	266.56
52972	ECP2 protein	GO:0003677 GO:0045892 GO:0046677	266.56
89258	Putative altered inheritance of mitochondria protein	-	263.34

**Table 6:** Top-10 most expressed specific genes and effectors in *P. fijiensis* (conidial transcriptome).

**ID Gene**	**NCBI Description**	**GO Term**	**RPKM**
Pfijiensis_857	No hit	-	216.41
Pfijiensis_645	No hit	-	215.34
Pfijiensis_2011	No hit	-	211.18
Pfijiensis_1733	No hit	-	209.16
Pfijiensis_887	No hit	-	190.89
Pfijiensis_944	No hit	-	188.43
Pfijiensis_1491	No hit	-	187.62
Pfijiensis_1874	No hit	-	180.64
Pfijiensis_424	No hit	-	174.16
Pfijiensis_1096	No hit	-	173.47
**JGI ID**	**NCBI description**	**GO term**	**RPKM**
Pfijiensis_2311	No hit	-	105.08
Pfijiensis_287	No hit	-	64.47

## Data Availability

The *P. fijiensis* conidial transcriptome data are available in the NCBI database with Sequence Read Archive (SRA) accession SRR25947114, released on 14 September 2023.

## Supplementary Materials

The following supporting information can be downloaded at: https://www.mdpi.com/article/10.3390/jof9100970/s1. Table S1. *Pseudocercospora fijiensis* conidial transcripts characterization; Table S2. Important processes usually found in conidia; Table S3. *Pseudocercospora fijiensis* conidial homologs with PHI-database; Table S4. *Pseudocercospora fijiensis* conidial effector candidates.
